# Crowd psychology and the politics of co‐production: Social control, democratic order and the consequences of theory

**DOI:** 10.1111/bjso.70065

**Published:** 2026-03-12

**Authors:** Clifford Stott

**Affiliations:** ^1^ The Open University Law School Milton Keynes UK

**Keywords:** co‐production, crowd psychology, participatory action research, public order policing, social identity approach

## Abstract

Social psychology has long claimed neutrality in its explanations of collective behaviour, yet its foundational theories of crowds have repeatedly been co‐produced with institutions of authority and mobilized in the reactionary governance of social order. This article challenges the discipline's familiar origin myth—centred on benign laboratory demonstrations of social influence—by re‐situating crowd psychology as one of social psychology's earliest and most politically consequential points of emergence. From nineteenth‐century crowd theory, through mid‐twentieth‐century de‐individuation research, to contemporary public‐order doctrine, assumptions about the inherent irrationality and danger of collective action have been repeatedly reformulated in scientific form, their persistence reflecting institutional and ideological fit rather than explanatory adequacy. Against this background, the article repositions the Social Identity Approach and the Elaborated Social Identity Model (ESIM) not merely as theoretical corrections, but as a reorientation of how psychological knowledge is produced, authorized and used. Drawing on ethnographic participatory action research and sustained engagement with policing institutions in the United Kingdom, Europe, and the United States, it conceptualizes collective behaviour as interactional and normatively organized, with policing practices constitutive of crowd dynamics rather than external to them. The article argues that co‐production is not a methodological innovation but a historically persistent condition of social psychology and that the ESIM represents a distinctive attempt to govern this condition reflexively by redirecting psychological knowledge towards legitimacy, restraint and the facilitation of democratic rights. The broader implication is that social psychology cannot plausibly claim political neutrality: its concepts travel into institutions and practices, shaping how collective action is anticipated, governed and policed.

## INTRODUCTION

The often‐quoted claim that those who fail to learn from history are condemned to repeat it is usually attributed to Karl Marx but was in fact articulated by George Santayana (1905). Marx's own contribution, however, was to argue that history recurs not through accident, but because the structural conditions that shape action persist. For social psychology, this insight has particular force. Theories of social behaviour do not merely describe the social world; they enter into it, shaping institutional practices, legitimizing forms of intervention, and producing material consequences for those subject to them. Attending to history is therefore not simply a matter of intellectual perspective, but of responsibility.

This point is powerfully exemplified in the work of one of the discipline's most influential critical theorists, Michael Billig. In *More Examples, Less Theory*, Billig (2019) argues that psychology is dominated by positivist writing conventions that systematically strip away the human, historical, and political dimensions of social life. As a result, he suggests, ‘experimental reports can be example‐free, human‐free zones’ (p. 4). Such conventions do more than constrain explanation; they distance psychological knowledge from the people, practices, and institutions with which it is entangled. As Russell Spears, another highly influential social psychologist, once memorably remarked during a conference presentation, social psychology has become a discipline in which ‘the death of six million Jews during World War II is an anecdote in history, whilst a *p* value of less than .05 constitutes a fact’.

Billig's response is not a rejection of theory or method, but a call to re‐situate psychological knowledge historically, contextually, and politically. He argues for the use of carefully contextualized case studies that anchor analysis in real social worlds, and later extends this sensibility to statistics themselves, showing how they emerge within—and are mobilized for—particular political purposes (Billig & Marinho, 2025). Across these contributions, Billig foregrounds a central but often neglected issue: that psychologists have choices not only about how they analyse social phenomena, but about how their knowledge relates to power, practice and consequence.

Drawing on this insight, the present article departs deliberately from conventional academic writing—not as an indulgence in personal narrative, but because its central concern is with how social psychological knowledge about crowds has been historically produced in relation to power. The core claim is that social psychology has never been neutral with respect to institutions of authority: from classical crowd psychology through to de‐individuation theory and contemporary public‐order doctrine, theories of collective behaviour have been co‐produced with governance institutions and mobilized towards the reactionary control of social order. The problem is therefore not whether social psychology is political, but how its entanglement with power has been organized, legitimated and rendered invisible.

To develop this claim, the paper proceeds in three stages. It first re‐situates the origins of crowd psychology within nineteenth‐century projects of governance and social control, showing how early theories of collective behaviour were aligned with elite anxieties about disorder and authority. It then traces how these assumptions were experimentally recast in de‐individuation theory, stabilizing crowd pathology within the core assumptions of modern social psychology. Finally, it contrasts this lineage with the Social Identity Approach and the Elaborated Social Identity Model (ESIM), drawing on ethnographic and participatory action research with policing institutions in the United Kingdom, Europe, and the United States to show how co‐production can be reoriented towards legitimacy, facilitation, and the defence of democratic rights. The central argument is that co‐production is a historically persistent condition of social psychology—but one that can be governed in radically different ways, with profoundly different political and practical consequences.

## THE BIRTH OF CLASSICAL CROWD PSYCHOLOGY AND THE MYTH OF DISCIPLINARY ORIGINS

When I first encountered social psychology as an undergraduate, I was taught a familiar and apparently benign origin story. The discipline, we were told, began with Norman Triplett's (1898) study of cyclists, in which the presence of others appeared to enhance individual performance—a finding later formalized as ‘social facilitation’ (Zajonc, 1965). This experiment was presented to us as a foundational moment of social psychology: modest, empirical, and apolitical. Yet this narrative, endlessly reproduced elsewhere in textbooks and teaching, offers a profoundly misleading account not only of where the discipline originated, but of how psychological knowledge about collective behaviour has historically been produced and mobilized.

The problem is not that Triplett's study is unimportant (cf. Karau & Williams, 2017), but that its elevation to a sub‐disciplinary origin myth obscures a more complex and politically consequential history. Long before psychology emerged as a distinct academic field, scholars across Europe were grappling with the social and political consequences of industrialization, rapid urbanization, mass democracy, and recurrent revolutionary violence (McClelland, 1989). At the centre of these struggles lay an urgent and practical question: how could social order be maintained, authority asserted, and hierarchy preserved in societies in which the masses were becoming politically powerful through collective mobilization? The crowd, as both a material force and a symbolic threat, became a focal point through which elite anxieties about disorder, legitimacy, and control were articulated (Stott & Drury, 2016).

In this context, crowd theory can be understood as one of the earliest forms of social psychology. Rather than emerging at the margins of social thought, it lay at the heart of intellectual debates that would later crystallize into disciplines such as psychology, sociology, criminology, and political science. In the mid to late nineteenth century, however, these fields did not yet exist in their modern form, and early crowd theorists were not social psychologists in the contemporary sense, but historians, legal scholars, physicians, criminal anthropologists, lawyers and magistrates working within and around the formative social and behavioural sciences. Importantly, the knowledge they produced was not developed in dialogue with crowds as political actors, but in close alignment with projects of governance concerned with managing, containing, empowering or neutralizing collective action.

Central to these intellectual and political movements was the belief that social disorder could be rendered intelligible—and therefore governable—through scientific classification and measurement. Drawing on evolutionary biology, particularly Darwinian and Spencerian ideas, and on the emerging statistical techniques pioneered by figures such as Francis Galton, these thinkers sought to map social behaviour onto hierarchies of intelligence, morality, and civilization. In this sense, race theory was not a peripheral concern but a central organizing principle. Galton's application of statistics to human populations exemplified a broader project of ranking and regulating social groups, providing a scientific veneer for political assumptions about degeneration, inheritance, and social worth (Billig & Marinho, 2025). Within this framework, the crowd came to be understood as the visible manifestation of atavism: a regression to primitive psychological states triggered by collective proximity, hypnotic suggestion, and emotional contagion.

As is now well known, early crowd theory was ‘scientific’ less in the modern empirical sense than as a form of intellectual pseudo‐science. Its concepts were often speculative, its methods impressionistic, and its assumptions deeply entwined with the political and moral anxieties of the age. Yet this does not render it peripheral or trivial. On the contrary, classical crowd psychology constituted one of the earliest systematic attempts to theorize collective behaviour, authority, and social order. It provided a language through which the perceived dangers of mass politics could be named, analysed, and ultimately controlled. Crucially, it did so by locating the source of disorder not in inequality, repression or illegitimate authority, but in the psychology of the crowd itself—thereby displacing structural and political explanation in favour of psychological and anthropological diagnosis.

These ideas were never confined to abstract theorizing. From the outset, classical crowd psychology was entangled with concrete political and legal projects, and its influence flowed directly into practices of governance, control and legitimation. Enrico Ferri, a radical socialist lawyer and seminal figure in criminal anthropology, deployed crowd theory in court to argue that individuals involved in riots lacked full personal responsibility, using psychological accounts of collective influence to mitigate criminal culpability (Van Ginneken, 1992). Similarly, Scipio Sighele, essentially the founding father of crowd psychology, later became one of the most prominent intellectual figures associated with Italian fascism, where this theory was mobilized to justify authoritarian control and the suppression of dissent (ibid). Gustave Le Bon himself was explicit that his work was intended to instruct elites in how to manage, manipulate and govern the masses—a project that later readers, including authoritarian political leaders, would take up with enthusiasm (McClelland, 1989). These divergent political appropriations do not indicate misuse or distortion of crowd theory but rather testify to its power: its capacity to render collective action intelligible in ways that translated psychological explanation directly into political action.

From its inception, therefore, crowd psychology functioned not as a neutral science standing apart from politics, but as a form of knowledge co‐produced with institutions of authority and oriented towards the practical problem of social control. Social psychology as a formal sub‐discipline would emerge much later, but when it did, it inherited not only the conceptual vocabulary of classical crowd theory, but also its political commitments and practical consequences. When the field as we recognize it today began to take shape in the mid‐twentieth century, it did not do so on a blank slate. Instead, it absorbed a set of ideas about irrationality, contagion, anonymity and loss of self that had already been elaborated—and actively mobilized—decades earlier in nineteenth‐century debates about crime, revolution and the governance of public order.

To understand the origins of social psychology, therefore, requires more than a narrow focus on ostensibly neutral laboratory demonstrations of social facilitation at the turn of the twentieth century. It demands a confrontation with this earlier history, in which the crowd—and by implication sociality itself—was not an impartial empirical curiosity, but a central political problem of modernity, bound up with questions of authority, legitimacy and control. It is this pattern of knowledge production—where psychological explanation is aligned with governance rather than generated in dialogue with collective actors—that later experimental and ostensibly apolitical approaches would inherit, refine and stabilize in new scientific forms.

## FROM CLASSICAL CROWD PSYCHOLOGY TO DE‐INDIVIDUATION: THE EXPERIMENTAL RECASTING OF COLLECTIVE PATHOLOGY

Perhaps the first recognizably modern social psychology textbook was published by Floyd Allport during the inter‐war period, at a time when behaviourism exerted a dominant influence over American psychology (Allport, 1924). Allport's formulation was explicitly individualistic, defining social psychology as the study of how the presence of others influences individual behaviour. In doing so, it helped establish a laboratory‐based, positivist orientation that would later become disciplinary orthodoxy. Around the same period, Muzafer Sherif produced his influential work on the formation of group norms, most notably demonstrating that perceptual judgements could be socially constructed rather than merely individually determined (Sherif, 1936). While Sherif's findings pointed towards the social regulation of perception, they were nevertheless incorporated within an experimental framework that prioritized controlled settings, abstracted tasks and the isolation of psychological mechanisms.

What is significant about this transition is not simply the emergence of experimentation, but the way it reconfigured the relationship between psychological knowledge and collective action. Questions that had previously been posed in explicitly political and institutional terms—concerning authority, legitimacy and social order—were increasingly reformulated as problems of individual cognition and behaviour. Collective actors were no longer engaged as participants in historically situated conflicts but transformed into passive experimental subjects, their actions abstracted from the social conditions in which they occurred.

It was in the aftermath of the Second World War that social psychology consolidated as the discipline we broadly recognize today. The catastrophic violence of the mid‐twentieth century rendered questions of mass behaviour, conformity, obedience, prejudice and intergroup conflict intellectually urgent. Post‐war social psychology took these issues as central concerns and developed a research agenda that sought to address them through systematic experimentation. Programmes of work on conformity (Asch, 1951), obedience to authority (Milgram, 1963), and prejudice and intergroup relations (Allport, 1954; Sherif & Sherif, 1953) came to define the field. In important respects, this body of research marked a departure from the most restrictive forms of behaviourism by reintroducing theoretical constructs such as attitudes, beliefs, norms, intentions and perceptions as mediating processes in the explanation of behaviour.

At the same time, experimental methods and increasingly sophisticated statistical techniques became the privileged means through which social processes were examined, promising rigour, objectivity and generalizability. This methodological consolidation did not simply refine how social behaviour was studied; it also shaped what counted as plausible explanation. As experimental social psychology increasingly prioritized abstraction, control and replicability, accounts of mass violence and social disorder were progressively relocated from their historical, political and institutional conditions to psychological universals operating at the level of the individual (Haslam & McGarty, 2001).

This explanatory orientation was stabilized through the emerging institutional infrastructures of the discipline. One of the earliest and most influential publication outlets for social psychological research in the United States was *The Journal of Abnormal and Social Psychology*—a title that reflected, and helped to normalize, the treatment of collective phenomena through the lens of deviance and disorder. Within this framework, collective actors were positioned primarily as objects of diagnosis and explanation rather than as participants in the production of social knowledge. It was through this combined process of methodological abstraction and institutional framing that social psychology distanced itself from overtly political explanation while, paradoxically, reproducing many of the core ideological assumptions of earlier crowd psychology in a newly scientific form.

A key moment in this transition can be found in the work of Leon Festinger—one of the most influential figures in twentieth‐century social psychology—and his colleagues. In *Some Consequences of De‐individuation in a Group* (Festinger et al., 1952), they proposed that anonymity and reduced identifiability within groups weakened internal restraints, thereby increasing the likelihood of antisocial behaviour. Although framed as an experimental investigation of group cohesion, the logic of de‐individuation closely echoed earlier assumptions of classical crowd theory. Once again, the collective was positioned as an external force that reliably produced a psychological condition in which individuality dissolved, moral regulation weakened and behaviour regressed towards more primitive or atavistic forms.

What distinguished Festinger's intervention from classical crowd theorists such as Le Bon or Sighele was not the substance of these assumptions, but their methodological translation. De‐individuation—a term coined by Festinger and his colleagues—was effectively Le Bon's anonymity under another name but crucially, it was operationalized as an independent variable: a taken‐for‐granted condition through which disinhibition was assumed to arise. The study was not, in practice, an investigation of de‐individuation itself, but an exploration of whether the presumed disinhibition and antisocial tendencies associated with group contexts could serve as the basis for psychological cohesion. In this way, inherited assumptions about collective pathology were embedded within the emerging experimental core of the discipline and re‐endowed with scientific authority.

This development illustrates how deeply classical crowd theory had already become sedimented within social theory. The historically situated crowd—defined by political conflict, power relations and institutional response—was gradually replaced by the abstracted, decontextualized group, operationalized through laboratory manipulations of presence, anonymity and identifiability. The pathological consequences of being in a group were not treated as a contested hypothesis, but as an implicit starting point for experimental inquiry. Early experimental social psychology thus functioned as a stabilizing mechanism through which past political assumptions about collective danger were normalized, routinized and insulated from historical critique.

As noted above, this work on de‐individuation sat alongside parallel lines of foundational research into conformity, obedience and intergroup hostility that came to prominence in the 1950s and 1960s, all of which shared a concern with the apparent fragility of individual autonomy and rationality in social contexts. Stanley Milgram's studies of obedience to authority are emblematic in this respect (Milgram, 1963). Subsequent critical re‐analyses have demonstrated how Milgram's complex and variable findings were obscured by an analytic focus on a single experimental variation, reinforcing the false impression of a general human tendency towards obedience while masking substantial resistance and dissent (Gibson, 2013; Haslam et al., 2014; Reicher & Haslam, 2011). Milgram's later theoretical appeal to the ‘agentic state’ resonated closely with earlier ideas of loss of self‐control and moral dissolution under external social influence (Milgram, 1970).

In this respect, there is an evident continuity between classical crowd psychology and early modern social psychology. While the overt language of degeneration, race and atavism receded, the underlying model of collective behaviour remained strikingly familiar. Groups continued to be understood as sites of psychological loss rather than social meaning; collectivity was associated with diminished rationality and moral control; and the sources of conflict were located within the psychology of participants rather than in the structures of authority, inequality or legitimacy that shaped their actions. Through experimental abstraction, social psychology was able to present itself as a progressive, value‐neutral science, even as it reproduced long‐standing assumptions about the dangers of collective action—assumptions that would once again be mobilized to explain and justify coercive responses to crowds and social movements.

## STRUCTURAL RECURRENCE AND THE SCIENTIFIC RECASTING OF THE CROWD IN 1960S AMERICA

What is striking in retrospect is not simply the persistence of ideas about collective pathology, but the historical conditions under which they are repeatedly reasserted. As with the birth of classical crowd psychology in nineteenth‐century Europe, the consolidation and amplification of de‐individuation theory in the mid‐twentieth century coincided with a profound crisis of social order and political authority. In both moments, rapid social change, mass mobilization and challenges to established hierarchies rendered the crowd newly visible as a problem demanding explanation and control. Crowd psychology did not flourish in periods of relative stability; it gained traction precisely when authority, legitimacy and governance were under pressure. Seen in this light, the resurgence of crowd pathology within modern social psychology should be understood not as abstract theoretical innovation, but as a historically recurrent intellectual response to moments of social and political crisis.

This pattern is particularly evident in the social and political landscape of the United States during the 1960s. Across the decade, American society was marked by sustained civil unrest, with waves of urban disorder occurring in hundreds of cities (National Advisory Commission on Civil Disorders, 1968). These disturbances were closely intertwined with structural racism, the civil rights movement, homophobia, opposition to the Vietnam War and broader challenges to racial, generational and political hierarchies (Armstrong & Crage, 2006; Gitlin, 1987; McAdam, 1982). At the same time, mass gatherings associated with the counterculture—from large‐scale anti‐war demonstrations to events such as the Woodstock music festival in 1969—placed unprecedented numbers of people into collective spaces beyond the direct control of traditional authorities (Marwick, 1998; Roszak, 1969). The crowd emerged simultaneously as a symbol of democratic possibility and as a perceived threat to social and moral hegemony. As in earlier historical moments, collective action became a focal point through which anxieties about disorder, legitimacy and control were articulated.

It was within this climate of sustained unrest, contested authority and heightened concern with social control that Philip Zimbardo's work on de‐individuation emerged and rapidly gained prominence. First presented at the 1969 *Nebraska Symposium on Motivation* and published shortly thereafter, his analysis of de‐individuated aggression quickly acquired canonical status within social psychology, routinely positioned alongside Milgram's obedience studies and Asch's conformity paradigm as a defining contribution of the period (Zimbardo, 1970). Yet the authority of this study should not be understood in terms of methodological innovation or theoretical novelty, for it offered neither. Rather, its influence lay in the way it crystallized, dramatized and endowed with scientific legitimacy a set of assumptions about collective behaviour that were already deeply embedded within contemporary social, political and institutional thought (Reicher & Haslam, 2006).

Extending Festinger's earlier formulation, Zimbardo's account treated anonymity, immersion in a group, and reduced identifiability as conditions capable of precipitating profound moral transformation. Under de‐individuated conditions, individuals were portrayed as relinquishing self‐regulation, internalized norms and ethical restraint, becoming capable of acts they would otherwise find unthinkable. Despite the availability of contradictory evidence demonstrating that anonymity can amplify norm‐consistent behaviour rather than disinhibition (Johnson & Downing, 1979; Postmes & Spears, 1998), these assumptions were taken up with little sustained critical scrutiny. Drawing on experimental techniques already controversial in the wake of Milgram's shock paradigm—relying on deception, induced distress and simulated violence—Zimbardo's work achieved its authority less through cumulative empirical validation than through its pedagogical force and rhetorical power. Although the Nebraska Symposium paper was not subject to conventional peer review, nor embedded within a programme of replication or theoretical refinement, its claims were rapidly institutionalized and taught for decades as robust evidence of the dangers inherent in collective anonymity.

Such uncritical receptiveness cannot be explained by evidential rigour alone. Rather, the appeal of de‐individuation theory is intelligible only when situated within the wider social context in which it circulated. At the end of a decade marked by civil unrest, mass protest and escalating confrontations between crowds and authorities, de‐individuation offered a compelling explanation for disorder that did not require recourse to structural inequality, political repression or failures of legitimacy. Collective violence could instead be understood as the predictable outcome of situational anonymity and group immersion. In this sense, Zimbardo's work functioned less as the discovery of new psychological processes than as a scientific articulation of a widely shared common sense: an ideological assumption that located the source of disorder within the psychology of crowds rather than within the conditions of their governance.

Crucially, this scientific articulation carried practical consequences. By framing collective transgression as the product of situational anonymity rather than political grievance or social injustice, de‐individuation theory aligned readily with US doctrines of crowd control. The implication was not merely that crowds could become dangerous, but that danger was inherent to collectivity itself. Under such a model, preventative surveillance, pre‐emptive dispersal and coercive containment could be justified as rational responses to an underlying psychological pathology. Experimental social psychology thus functioned not only as an explanation, but as legitimation—providing authoritative language through which control could be rendered necessary and proportionate.

Zimbardo's later work—most notably the Stanford Prison Experiment—would intensify these themes and ultimately provoke widespread ethical controversy, contributing to the development of more restrictive governance of psychological experimentation. Yet this institutional reckoning came only after de‐individuation theory had already achieved disciplinary authority and begun to circulate well beyond the laboratory. Its significance therefore lies not primarily in its empirical adequacy, but in its role in amplifying and legitimizing a historically recurrent model of the crowd—one that positioned collective action as a pathological intrusion into social order and rendered its control both necessary and scientifically justified (Haslam et al. 2019).

Seen in this light, the resurgence of crowd pathology within modern social psychology should not be understood as a neutral scientific response to empirical problems, but as a patterned intellectual reaction to moments of social crisis. Experimental positivism did not correct the assumptions of classical crowd theory; it stabilized them. By translating historically and politically charged ideas into the language of value‐neutral experimentation, social psychology rendered them authoritative, durable and readily transferable into institutional practice. The consequences of this move would extend far beyond the academy, shaping how collective action was anticipated, interpreted and policed in the decades that followed.

## MOMBOISSE AND CROWD PSYCHOLOGY AS A TECHNOLOGY OF SOCIAL CONTROL

By the late 1960s, social psychology was only one of several intellectual currents contributing to the construction of the crowd as a pathological social form. Long before the concept of de‐individuation was formalized, similar assumptions were already well established within American sociology, legal thought and policing practice. Early sociological analyses of collective behaviour, associated with figures such as Blumer and Lohman, framed crowds as emotionally volatile, weakly norm‐governed and prone to escalation (Blumer, 1951; Lohman, 1947). Although emerging from different theoretical traditions, these accounts shared a clear lineage with classical crowd psychology, including a common emphasis on contagion, loss of restraint and the dissolution of individuality. As Schweingruber (2000) argues, such ideas circulated widely across disciplinary and institutional boundaries, forming a tradition of ‘mob sociology’ that profoundly shaped how collective action was understood and governed.

Raymond Momboisse's work occupies a pivotal position within this intellectual and institutional landscape. Published at the height of the civil unrest in the United States, *Riots, Revolts and Insurrections* (Momboisse, 1967) appeared immediately prior to the report of the National Advisory Commission on Civil Disorders—the so‐called Kerner Commission. Established by President Lyndon B. Johnson in response to the widespread urban uprisings, the Commission concluded that the primary causes of disorder lay not with agitators, but in entrenched racial inequality, economic exclusion and the systematic failure of political and policing institutions (National Advisory Commission on Civil Disorders, 1968). Crucially, however, while the Commission rejected conspiratorial accounts of unrest, it did not fundamentally displace prevailing psychological assumptions about crowds themselves, nor did it offer an alternative framework for understanding collective behaviour in action.

Momboisse did not present himself as a social scientist advancing new theoretical insights into crowd behaviour. Rather, he wrote as a practitioner‐expert, drawing on his institutional authority as Deputy Attorney General for the State of California and his advisory roles within national debates on riot control and law enforcement. Like Le Bon before him, his significance lies not in theoretical innovation, but in the way his work consolidated and operationalized a set of assumptions about crowds that had already become deeply embedded within social thought—assumptions that stood in marked tension with the structural diagnosis advanced by the Kerner Commission. Crowd psychology here did not function as a detached explanation but as an organizing framework for co‐produced action oriented towards control and containment.

As Schweingruber (2000) notes, Momboisse's account of the mob closely recapitulates the core propositions of classical crowd theory and early sociological crowd analysis—irrationality, emotional contagion, loss of individuality and an inherent propensity for violence—often without explicit citation. He argues that while this absence of attribution might be read as a failure of scholarly rigour, it is better understood as evidence of sedimentation: by the late 1960s, these assumptions no longer required defence. They had become common sense, circulating seamlessly between sociology, psychology, law and policing and thereby acquiring a taken‐for‐granted authority that rendered them difficult to contest.

This status is evident throughout Momboisse's own language. Individuals are portrayed as subsumed by a collective mentality in which rationality and moral restraint are overwhelmed: ‘The individual is under the domination of the mob mind, the domination of low‐intelligence people, the domination of lower instincts and emotions’ (Momboisse, 1967, p. 17). Crowd behaviour is characterized as fundamentally irrational, governed ‘not by intellect, but by passion; not by cool calculation, but by impulse’ (p. 30). Under conditions of collective excitement, people are described as highly suggestible, ‘willing to follow uncritically the lead of a demagogue or even some rabid mob member’ (p. 386), becoming ‘citizens under control of forces beyond them’ who, once ‘sanity returns,’ are ‘unable to explain why they did what they are doing’ (p. 393).

Momboisse also deploys a quasi‐physiological imagery reminiscent of nineteenth‐century contagion theory. Disorder is depicted as spreading through the body politic as if by electrical transmission: ‘It seems as if some electric current flows through the veins of all citizens and leads them at once to rejection of all law’ (p. 19). Within this framework, violence is not understood as a response to grievance or illegitimacy, but as the consequence of weakened authority. Any ‘apparent weakening in the strength or attitude of the forces of law and order…encourages violence’ (p. 19). Disorder is thus rendered a psychological condition to be suppressed rather than a political problem to be addressed.

What distinguishes Momboisse's contribution is the scope and purpose of its application. *Riots, Revolts and Insurrections* is not merely a recapitulation of mob psychology; it is a comprehensive manual of governance. It sets out an integrated doctrine of control, encompassing planning, intelligence, command structures, logistics, tactical formations, stakeholder responsibility, gubernatorial authority and the use of force. Crowd psychology functions here as the organizing logic through which public order is imagined and managed, translating directly into prescriptions for intervention: ‘Police must interfere with the milling and rumour process; the crowd must be dispersed’ (p. 392). Psychological explanation is thus explicitly oriented towards the maintenance of order through containment and control.

The historical timing of this doctrinal consolidation is critical. Much of the diffusion of mob sociology and psychology into American policing occurred before the development of constitutional case law that currently constrains municipal and state police action through the First, Fourth and Fourteenth Amendments. At the point when Momboisse's doctrines were being formulated and institutionalized, police discretion was expansive and judicial oversight limited (Schweingruber, 2000). This dynamic intensified in the wake of the Kent State shootings in 1970. Rather than prompting a fundamental reassessment of crowd psychology, the tragedy accelerated demands for professionalization, coordination and standardization of police responses to collective disorder (President's Commission on Campus Unrest, 1970). It was through this process of reform and standardization—rather than academic debate—that Momboisse's framework achieved its most enduring influence.

Crowd psychology did not disappear under this reform; it was evidently institutionalized within it. Indeed, the assumptions first articulated by Momboisse and then by Zimbardo continue to inform contemporary public‐order doctrine in the United States, including their explicit incorporation into the chapter on crowd psychology in the *Field Force Operations Student Guide* (FEMA, 2016) a key national guidance framework for public order policing in the US. What is presented as modern, evidence‐based practice thus rests on a model of collective behaviour whose intellectual origins stretch back more than a century.

Seen in this light, Momboisse's and Zimbardo's work should not be understood as outliers or historical excess. They both represent the logical outcome of a long intellectual trajectory in which psychological and sociological knowledge about crowds was repeatedly co‐produced with institutions of authority for the purpose of repression and control, while presenting itself as neutral expertise. Experimental social psychology played an important role in legitimizing this trajectory by supplying scientific language and authority. But it was through legal, political and policing institutions that these ideas acquired material force.

## RECONTEXTUALIZING THE CROWD: SOCIAL IDENTITY, NORMATIVE CHANGE AND THE REORIENTATION OF SOCIAL PSYCHOLOGY

This history matters not because social psychology was co‐produced with authority per se—such collaboration is arguably unavoidable—but because the purposes of that co‐production became concealed and remain under examined. Crowd psychology did not merely describe collective action; it helped define what collective action was for, whose interests it threatened and how it should be governed. Evaluating its legacy therefore requires attention not only to theoretical validity, but to the purposes, institutional alignments and power relations that psychological knowledge has historically served. It is against this background that later efforts to re‐orient co‐production towards democratic facilitation, legitimacy and the protection of rights must be understood.

The theoretical foundations for a reorientation of crowd and social psychology were already being laid within European social psychology in the late 1970s and early 1980s. Led most prominently by Henri Tajfel, the *Social Identity Approach* emerged as an explicit critique of the decontextualized and reductionist tendencies that had come to dominate American experimental social psychology. Tajfel's intervention was not simply a refinement of cognitive theory, but a reconceptualization of the self as socially and historically constituted. Social identity theory treated the self as multiple and relational, embedded within structures of power, inequality and legitimacy. In this sense, it was not merely a theory of cognition, but a theory of society and social relations—one that reasserted the qualitative distinctiveness of group‐level processes and their irreducibility to individual psychology (Ellemers & Haslam, 2012).

This theoretical move was further elaborated through Self‐Categorization Theory, which provided a dynamic account of how social identities become cognitively salient, how their content is shaped by social context and how collective norms emerge from shared categorization rather than from anonymity or loss of self (Turner & Reynolds, 2012). Together, these approaches directly challenged the core assumptions of classical crowd psychology and its experimental reformulations. The crowd and the social group were no longer understood as sites of psychological regression, but as normatively organized collectives whose behaviour could only be understood in relation to intergroup dynamics and relations with authority.

Importantly, this reorientation did not emerge in isolation. It can be situated within a longer, partially obscured intellectual tradition associated with Kurt Lewin, whose work on group dynamics and *Action Research* in the immediate aftermath of the Second World War had already rejected the separation of theory from practice. Lewin's oft‐cited injunction that ‘there is nothing so practical as a good theory’ was not a call for instrumental application alone, but for a form of social psychology explicitly oriented towards ethical purpose. Writing in the shadow of fascism and genocide, Lewin argued that the task of social science was not merely to explain social processes, but to use theory to intervene in them in ways that advanced democratic values, reduced conflict and transformed relations of power—to do good things with social psychological theory (Lewin, 1946, 1947).

Lewin's field theory—encapsulated in the proposition that behaviour is a function of the person and the environment—anticipated many of the later critiques of reductionism advanced by the Social Identity tradition. Groups were understood as dynamic systems rather than aggregates of individuals; authority relations as constitutive of behaviour rather than external constraints and social change as a function of shifts in norms, identities and power rather than irrational psychological states (Lewin, 1951). Crucially, Lewin's approach treated collaboration with institutions of power and authority as unavoidable. The central question was not whether social psychology should engage with such institutions, but to what ends that engagement would be directed.

Within the social identity framework, a key problem for understanding crowds was how collective norms emerge, stabilize and transform through interaction—particularly through encounters with authority (Reicher, 1987). This problem marked a decisive break with classical and de‐individuation‐based accounts. It also rendered policing analytically central rather than peripheral. Crowd behaviour could not be explained independently of how police categorized, anticipated and acted towards collective actors. Authority was not an external influence on the crowd, but a constitutive element in the production of collective action itself (Reicher, 1996; Stott & Reicher, 1998a).

These theoretical commitments carried clear methodological implications. If collective behaviour is normatively structured, relational and historically situated, then it cannot be adequately studied through abstracted laboratory simulations alone. Instead, it requires methods capable of capturing interaction as it unfolds in real social settings, including the practices through which authority is exercised and legitimacy contested. Observational, ethnographic and participatory approaches therefore followed directly from this framework. They were not departures from social psychology orthodoxy but attempts to realize its critical and ethical potential by situating psychological processes within the social fields in which they are enacted (Drury & Stott, 2001; c.f. Reicher & Hopkins, 2001).

Research informed by this perspective, ultimately reliant upon co‐production with both crowd participants and police, repeatedly demonstrated that collective conflict was rarely the product of shared intent or intrinsic crowd volatility. Rather, escalation often emerged through patterns of interaction in which policing practices—guided by assumptions derived from classical crowd theory—misrecognized crowds and acted towards them as inherently and uniformly dangerous (Stott & Reicher, 1998a; Drury et al., 2003). Such interventions altered the social context faced by crowd participants, undermined perceptions of legitimacy and produced precisely the forms of disorder they were intended to prevent (Drury & Reicher, 2000; Reicher, 1996; Stott & Drury, 2000). On this account, crowd disorder is best understood as an emergent and relational process, generated through asymmetric power relations enacted in institutional practice.

It was within this intellectual and ethical space that the Elaborated Social Identity Model (ESIM) was developed (Drury, 2025). Building on Social Identity Theory and Self‐Categorization Theory, ESIM provided a framework for explaining how identity change and collective conflict emerge through dynamic intergroup relations rather than through psychological loss of control. ESIM conceptualized crowd behaviour as normatively organized and historically situated, with collective action shaped by the unfolding relationship between crowd participants and authorities, particularly policing actors. Crucially, it demonstrated that shifts in crowd norms are not intrinsic to collectivity, but arise through processes of categorization, legitimacy and interaction.

This insight raises a more fundamental question about the role of psychological theory itself. If theories of collective behaviour are not merely descriptive but actively implicated in shaping institutional action—and if those actions can escalate conflict or suppress legitimate dissent—then social psychology cannot plausibly claim political neutrality. The task of the discipline cannot merely be confined to detached explanation or retrospective critique. Rather, it must engage directly with the purposes and consequences of its knowledge as it is translated into practice. From this perspective, the challenge is no longer simply to explain crowds but to develop forms of psychological knowledge capable of transforming how collective action is understood, governed and policed.

It was in the empirical study of football hooliganism that these theoretical and ethical commitments were first translated into sustained practice, marking the emergence of an explicitly co‐produced model of policing oriented not towards repression, but towards legitimacy, facilitation and the protection of collective rights.

## ESIM, FOOTBALL HOOLIGANISM AND THE EMERGENCE OF PURPOSIVE CO‐PRODUCED POLICING

The early empirical foundations of ESIM were established, in part, through a programme of ethnographic research on football crowd disorder conducted during major international tournaments, most notably Italia 90 and France 98 (Stott & Reicher, 1998b; Stott et al., 2001). These studies demonstrated that collective ‘disorder’ was not inevitable but could emerge contingently through patterns of interaction between crowds and police. Where policing practices categorized crowd participants indiscriminately and acted in ways perceived as illegitimate, previously heterogeneous gatherings could come to redefine themselves in oppositional terms, producing precisely the forms of collective confrontation that classical crowd theory assumed to be intrinsic (Pearson & Stott, 2022; Stott & Maguire, 2026).

At the time, however, the significance of these findings lay primarily in their explanatory force rather than their practical influence. Despite their theoretical and empirical contribution, early attempts to translate ESIM insights into operational change encountered substantial institutional resistance within the United Kingdom. Contemporary social psychology remained peripheral to policing and security practice, and public‐order doctrine continued to be shaped by assumptions derived from classical crowd theory and its experimental reformulations. In this initial phase, the football research functioned largely as critique: it exposed the limitations and unintended consequences of existing approaches but did not yet reshape how policing was organized or enacted.

Football nonetheless proved to be a critical site for the subsequent development of co‐produced policing. This was not because football crowds were uniquely prone to disorder, but because football policing was unusually visible, politically sensitive and institutionally entrenched. Events such as the Heysel Stadium disaster in 1985, episodes of serious violence at subsequent international tournaments and the Hillsborough disaster in 1989 placed policing practices under sustained scrutiny from governments, the media and transnational bodies. These moments created conditions in which failures of public‐order management could no longer be contained, normalized or denied. Football thus became a domain in which psychological theory had an opportunity to engage directly with authority, rather than remaining confined to retrospective explanation.

This engagement was catalysed decisively by the events surrounding UEFA Euro 2000. Highly publicized disorder involving England supporters, alongside sustained international criticism, generated a significant political and institutional crisis for the newly elected Labour government. In response, the UK Home Office tasked its public‐order unit with conducting a formal inquiry into football disorder and crowd management practices. It was through this process—driven by political accountability rather than academic persuasion—that ESIM‐based research was first drawn directly into policy deliberation and operational planning.

At the same time, Euro 2000 opened pathways for international academic‐police collaboration that proved crucial to the reorientation of co‐production. Engagement with the Dutch Police Academy and, most importantly, with the Portuguese police created opportunities not merely for dialogue and joint observation, but for the prospective application of ESIM‐derived principles to the design of policing operations. These engagements occurred against the backdrop of Portugal's preparation to host the 2004 European Championship, which placed public‐order policing and international scrutiny high on the agenda of senior police leaders. The forthcoming tournament created both urgency and institutional incentive to address the security challenges that ESIM‐based research was directly concerned with. In this context, ESIM‐informed approaches were taken up in a systematic and ambitious manner. Rather than being trialled in isolated contexts, they were implemented across all major host cities, shaping national policing strategy for the tournament as a whole.

This marked a decisive shift from explanation to intervention. In Portugal, ESIM was not merely used retrospectively to account for disorder, but prospectively to design policing practices aimed at preventing escalation by building legitimacy, differentiating between groups and facilitating lawful collective behaviour. Crucially, this collaboration did not operate on the assumption of a simple knowledge deficit. Many of the principles involved were already recognized by a cohort of progressive police leaders who were seeking to move away from containment‐oriented reactionary models of public order. The value of co‐production lay in its capacity to provide these actors with a coherent theoretical framework, empirical warrant and external legitimacy through which an alternative approach could be articulated, defended and sustained. In this sense, the collaboration reshaped the internal politics of policing as much as operational practice itself. While initial scepticism among senior leadership remained—reflecting the entrenched influence of classical crowd theory, the historical legacy of Portugal's former totalitarian state and concerns about the risks of avoiding restraint—the unfolding of the tournament rendered such resistance increasingly difficult to sustain. Levels of conflict were low, large‐scale disorder was avoided, and police–crowd relations were widely regarded as successful (Stott, 2020).

The significance of this episode lies not simply in its immediate outcomes, but in what it demonstrated institutionally. For the first time, ESIM functioned as a viable alternative doctrine for public‐order policing, capable of operating at a national scale under conditions of intense political and operational pressure. Crucially, this did not involve a withdrawal from engagement with authority, but a re‐specification of what that engagement was for. Co‐production remained firmly located within institutions of power, yet its purpose shifted from containment and repression towards facilitation, legitimacy and the protection of collective rights.

On the basis of its success, the Portuguese approach was subsequently promoted at the international level and woven into European policing policy. ESIM‐informed principles went on to shape security arrangements for later major tournaments, including the European Championships in Austria/Switzerland and Poland/Ukraine, and continue to be promoted within UEFA as the overarching conceptual framework for crowd management, even if implementation varies across jurisdictions. Importantly, this diffusion was accompanied by further academic evaluation and UKRI—particularly ESRC—funded research, which consolidated the evidential base of ESIM‐derived policing models and extended their application (Stott et al., 2007, 2008).

Consequently, by the time later crises emerged an alternative framework for understanding and policing crowds was no longer merely theoretical. Through a framework of co‐production, it was empirically grounded, operationally tested and institutionally articulated. In this sense, the work on football hooliganism did not simply generate knowledge; it created the conditions of readiness through which moments of crisis could be leveraged for further meaningful reform. The football case thus represents the first sustained demonstration that social psychology could engage in explicit co‐production with authority while re‐orienting that collaboration towards democratic ends.

## CRISIS, LEGITIMACY AND THE FORMALIZATION OF CO‐PRODUCED KNOWLEDGE: THE DEATH OF IAN TOMLINSON

The death of Ian Tomlinson during the G20 protests in London in April 2009 marked a decisive rupture in British public‐order policing. Unlike earlier moments of controversy or disorder, this event generated a fundamental crisis of legitimacy. Tomlinson was not a protester engaged in confrontation, but a member of the public attempting to leave the area. His death—captured on video and widely disseminated—exposed a profound disjuncture between police assumptions about crowd threat and the lived reality of collective events. In doing so, it rendered visible the human cost of a policing model still shaped, both explicitly and institutionally, by the assumptions of classical crowd psychology.

At the centre of this model lay a familiar and formally articulated logic. Crowds were treated as inherently volatile, prone to irrational escalation, and therefore requiring decisive, pre‐emptive control. These assumptions were not merely residual or implicit. Classical crowd theory—including the work of Le Bon—continued to be taught within British public‐order training and used as a reference point against which commanders' understanding of crowd dynamics was assessed. Tactical responses accordingly prioritized containment, coercive presence and the rapid suppression of perceived disorder. The circumstances of Tomlinson's death demonstrated with tragic clarity how such theoretically grounded assumptions could translate into systematic misrecognition of threat, disproportionate use of force and the escalation of harm.

The subsequent inquiry by Her Majesty's Inspectorate of Constabulary (HMIC) constituted a critical moment of institutional reflexivity. Crucially, HMIC did not merely call for procedural reform or improved training. It explicitly engaged with psychological theory, rejected classical crowd psychology as an adequate basis for public‐order policing and recommended its replacement with an interactional, legitimacy‐based understanding of crowd dynamics grounded in ESIM (HMIC, 2009). This marked the first formal acknowledgement by the British state that prevailing psychological assumptions about crowds were not simply outdated, but actively contributing to operational failure and loss of life.

What matters for the argument developed in this paper is not only the content of HMIC's recommendations, but the conditions under which they became possible. The inquiry did not generate a new body of psychological knowledge. Rather, it created an institutional opening in which alternative forms of knowledge—developed elsewhere, under different political conditions—could acquire authoritative force. Research on football policing, produced through sustained observation, ethnography and long‐term collaboration with police organizations, had already demonstrated that crowd behaviour was interactional, normatively structured and highly sensitive to policing style. When crisis struck, this work was available, intelligible and—crucially—trusted by practitioners.

This is where the concept of readiness becomes analytically central. The influence of this research did not stem from superior epistemic certainty in a positivist sense, nor from dominance within academic debate. Much of its authority derived from knowledge products that fell outside conventional academic reward structures: internal reports, operational briefings, practitioner‐focused analyses and iterative engagement with policing organizations. These forms of knowledge were valued not because they abstracted from practice, but because they were produced in dialogue with it and demonstrably capable of anticipating risk, reducing conflict and restoring legitimacy.

It was in this context that participatory action research (PAR) came to be recognized as a methodological logic already implicit in the research practice. Through sustained ethnographic engagement with policing institutions, it became increasingly clear that the work shared key features with PAR as articulated in other fields (Kemmis & McTaggart, 2005; Reason & Bradbury, 2008). Drawing on Lewin's insistence that valid social knowledge must be produced through engagement with real social problems, this approach went further by reconfiguring the relationship between researcher and practitioner. Rather than treating practitioners as recipients of expert knowledge, it recognized them as epistemic equals whose experiential, situational and institutional understanding constituted a vital form of social knowledge in its own right. The objective was no longer the production of theory alone, but the co‐production of knowledge products oriented towards operational change.

This shift had important practical consequences. Because practitioners were involved in the production of knowledge, rather than positioned as subjects of diagnosis or correction, the resulting frameworks carried greater legitimacy within policing organizations. Conversations about reform were faster, less defensive and less vulnerable to internal contestation. As with Portugal and Euro2004, co‐production functioned not as a mechanism for filling a knowledge deficit, but as a means of reshaping the internal politics of expertise—providing reform‐oriented actors with the conceptual resources, empirical warrant and institutional cover necessary to challenge more reactionary assumptions embedded within institutionalized UK public‐order doctrine.

Once these conditions were acknowledged, detached observation and post hoc critique became scientifically and ethically insufficient. Where policing practices were actively producing the dynamics they sought to prevent, knowledge production could no longer plausibly remain external to practice. Participatory action research (PAR) followed directly from the interactional ontology of ESIM and from the recognition that the consequences of theory were material and immediate. While epistemological certainty remained important, it was no longer the primary organizing principle of inquiry. The priority shifted towards whether psychological knowledge could intervene in practice in ways that reduced harm, prevented escalation and sustained legitimacy. The method did not precede the problem; it was demanded by it.

The Tomlinson case therefore crystallized a deeper rupture with positivist social psychology. The central question was no longer how to refine abstract explanations of crowd behaviour in pursuit of ever‐greater epistemic certainty, but how psychological knowledge could be mobilized to prevent harm and restore legitimacy in real‐world settings. This required a re‐evaluation of what counted as successful academic work. In this context, the most consequential outputs were not journal articles alone, but forms of knowledge capable of reshaping doctrine, training and operational decision‐making. Academic value was measured not primarily in citations, but in reduced conflict, enhanced legitimacy and the avoidance of tragedy.

Seen in this light, the death of Ian Tomlinson represents not only a failure of policing, but a failure of a particular model of social psychology—one that claimed political neutrality while remaining deeply implicated in the reproduction of coercive practice. The reforms that followed did not arise from theoretical debate alone, but from the convergence of crisis, institutional reflection and the availability of an alternative epistemological framework grounded in interaction, legitimacy and explicit co‐production. This moment did not end the influence of classical crowd theory, but it did render its assumptions contestable in practice and formalized a different way of producing psychological knowledge—one oriented not towards control, but towards democratic order.

## CRISIS, CO‐PRODUCTION AND DEMOCRATIC POLICING IN THE UNITED STATES: DIALOGUE POLICING AFTER 2020

The developments in the United States following 2020 represent the most explicit convergence of the historical, theoretical and methodological strands traced throughout this paper. As in earlier moments examined here—the emergence of classical crowd psychology in nineteenth‐century Europe, the consolidation of de‐individuation theory amid the unrest of the 1960s, and the reform of UK public‐order policing following the death of Ian Tomlinson—change was again catalysed not by academic debate alone, but by crisis. Once more, it was the legitimacy of policing, rather than the psychology of crowds per se, that became the central problem to be addressed.

The murder of George Floyd and the subsequent nationwide protests constituted the most extensive episode of civil unrest in the United States since the late 1960s. Demonstrations occurred in thousands of cities, often met with aggressive crowd‐control tactics, widespread use of force, mass arrests and escalating cycles of confrontation. Subsequently, in January 2021, a large crowd broke into the Capitol Hill building in Washington DC and disrupted the peaceful transfer of power, marking a qualitatively distinct challenge to democratic order and state authority (Haslam et al., 2023; Ntontis et al., 2024). Explanations of such disturbances frequently framed disorder in terms of crowd pathology, emotional contagion and loss of restraint. As noted above, such assumptions are deeply embedded within federal training, preparedness doctrine and emergency‐management frameworks that continue to bear the imprint of mob sociology (Federal Emergency Management Agency, 2016). Yet official reviews, court injunctions and independent investigations repeatedly demonstrated that policing responses often intensified conflict during the 2020 unrest, further eroding public trust and police legitimacy (e.g. Index Newspapers LLC v. City of Portland, 2020; Police Executive Research Forum, 2021; U.S. Department of Justice, 2023; American Civil Liberties Union, 2020).

It was within this context of acute political contestation and evident failures in crowd management that new opportunities for co‐production emerged. These did not arise because social psychology had resolved a theoretical debate—the dominance of ESIM within the academic literature was already well established—but because existing models of crowd control were failing in practice under conditions of heightened scrutiny, legal constraint and political pressure. As in earlier cases, crisis created an opening; but it did so only because alternative frameworks were already available, empirically grounded and capable of being translated into operational form.

The groundwork for this alternative had been laid through earlier European developments, particularly Dialogue Policing in Sweden and its subsequent adaptation within the United Kingdom through Police Liaison Teams (Stott et al., 2013). These initiatives had demonstrated that crowd conflict was not an inevitable feature of collective action, but an interactional outcome shaped by policing strategies, communication practices and perceived legitimacy. Crucially, they showed that ESIM was not merely an explanatory account of disorder, but a practical framework for designing policing interventions oriented towards facilitation, differentiation and restraint.

The City of Columbus, Ohio, became a sustained site in which these principles were translated into institutional practice in the United States (Stott et al., 2025). There, dialogue officers were embedded within protest operations as legitimacy‐building actors rather than negotiators in a narrow sense—tasked with communication, information‐gathering, facilitation and de‐escalation and integrated into command‐level decision‐making. This work involved a long‐term partnership between researchers, police leadership and operational officers, oriented explicitly towards dialogue‐based protest management rather than episodic reform.

More recently, this model has been taken up within an even more contested political terrain. In cities such as Portland, public‐order policing has become a focal point of struggle between federal and local authority, with protest policing situated at the intersection of constitutional rights, partisan conflict and competing models of governance. It is precisely in this environment—where federal crowd‐control doctrine continues to reproduce the assumptions of mob sociology and where local agencies face sustained political and legal pressure—that ESIM‐led dialogue policing has been co‐produced as a tool of democratic governance. Social psychology is not operating at a distance from this struggle, nor intervening retrospectively. It is engaged directly in the present tense, helping the municipal agency to design and stabilize policing practices that have evidently contributed to de‐escalation and the avoidance of a recurrence of the prolonged and severe unrest experienced in the city in 2020 (City of Portland, 2025; Price, 2025).

What is striking here is the temporal inversion of conventional academic impact. In these US cases, operational change has preceded formal academic publication. Dialogue structures, training practices and decision‐making frameworks are already in place; academic outputs follow later as documentation and historical reflection rather than as the linear drivers of reform. This is not a failure of dissemination, but a demonstration of relevance. Under conditions of crisis, legitimacy and political contestation, the value of social psychology lies not in epistemic priority, but in its capacity to contribute to timely, credible and operationally meaningful change.

What distinguishes these US cases is not simply the adoption of dialogue policing, but the depth of co‐production involved. I have been embedded within planning, deployment, observation and debrief processes, enabling ongoing feedback between theory, practice and outcome. Participatory action research has not appeared here as an ideological commitment or methodological preference, but as a practical necessity. Once it is recognized that police action helps constitute crowd dynamics, detached observation becomes analytically insufficient and ethically untenable. Knowledge capable of informing practice must be produced within the relations it seeks to shape.

The contrast with classical and de‐individuation‐based models of crowd psychology could not be clearer. Where mob sociology—institutionalized through figures such as Momboisse and sustained within federal emergency‐management doctrine—treats crowds as inherently dangerous and legitimates pre‐emptive control, ESIM‐derived dialogue policing begins from the assumption that crowds are normatively regulated and politically meaningful. Where older models justify coercion as necessary and inevitable, dialogue policing treats restraint, differentiation and facilitation as conditions of effectiveness. And where positivist social psychology sought epistemic certainty through abstraction, this work prioritizes consequential validity: whether theory, when enacted, reduces harm, sustains legitimacy and enables democratic protest.

Yet this transformation remains fragile. The uneven institutionalization of dialogue policing across thousands of autonomous police agencies in the US exposes a structural asymmetry at the heart of contemporary crowd governance. While mob sociology persists through centralized doctrine, standardized training and federal infrastructure, ESIM‐based approaches rely on local leadership, sustained co‐production and political will. The issue, therefore, is no longer one of psychological knowledge—ESIM is largely uncontested within the academic field—but of institutional architecture. What is lacking is a coherent structural framework capable of embedding democratic crowd psychology systematically within policing practice (Stott & Maguire, 2026).

Seen in this light, the contemporary US experience brings the paper full circle. Social psychology returns to the terrain from which it first emerged: the struggle to understand and govern collective action in moments of social upheaval. But it does so with a fundamentally different orientation. Rather than supplying pathologizing accounts that legitimate repression, ESIM‐led dialogue policing represents an effort to place social psychology in the service of democratic order—accepting that theory is always political, recognizing that knowledge travels through power and insisting that the purposes for which social psychology is co‐produced must therefore be confronted directly.

## CONCLUSION: CO‐PRODUCTION, POWER AND THE PURPOSES OF SOCIAL PSYCHOLOGY

This article has traced a recurring pattern in the history of social psychology: moments of social crisis give rise to theories of collective behaviour that are co‐produced with institutions of authority and mobilized in the governance of social order. In this respect, the tendency for history to repeat itself is not a matter of intellectual failure, but of structural continuity. Where the institutional conditions through which power is exercised remain unchanged, psychological knowledge is repeatedly drawn into similar roles. From classical crowd psychology, through de‐individuation theory, to contemporary emergency‐management doctrine, psychological accounts of crowds have repeatedly been oriented towards explaining disorder in ways that legitimate coercive intervention while obscuring questions of power, legitimacy and political grievance. The persistence of these models does not reflect superior explanatory adequacy, but their institutional and ideological fit with reactionary modes of governance.

Against this background, the contribution of the Social Identity Approach and the ESIM is not simply a matter of theoretical correction. Rather, it represents the possibility of a reorientation of how psychological knowledge is produced, for whom and to what ends. By conceptualizing collective behaviour as interactional, normatively organized and constituted through relations with authority, ESIM renders policing analytically central and exposes the consequences of theory as they unfold in practice. Crucially, it also makes visible what had previously remained concealed: that social psychology is always co‐produced with power, whether this is acknowledged or not.

What distinguishes the work traced in this paper is therefore not collaboration with authority per se, but the purposive redirection of that collaboration. Through ethnography, PAR, and sustained engagement with practitioners, co‐production was made explicit, reflexive and accountable. Knowledge was not produced to merely diagnose, but to facilitate legitimacy, restraint and the reduction of conflict through an emphasis on the protection and exercise of democratic rights. In this sense, the central shift is not epistemic but ethical and political: from a social psychology oriented towards repression to one oriented towards democratic order.

This does not imply, however, that ESIM represents a final or exhaustive framework for understanding collective action, nor that its practical application is immune from co‐option, misapplication or political distortion. Indeed, some would argue that by preventing conflict with the state, the ‘velvet glove in the iron fist’ undermines progressive social change. However, to be clear, the contribution of ESIM in this landscape is not that it is a panacea for social injustice or that it escapes structural inequalities and power. Rather, it is that the approach renders its own political orientation more explicit. In this case, the aim is to protect civil and human rights within democratic systems of power that retain stability and aim towards social justice through legitimacy and the rule of law. The programme of work is designed to de‐escalate conflict and criminality and, in doing so, to empower the freedom of peaceful assembly and expression, including political dissent. These are normative commitments rather than neutral outcomes, yet their convergence with ESIM is not the product of design. Rather, they coincide with the underlying identity‐based interactional dynamics that ESIM seeks to explain: the same principles appear to organize how crowd members often interpret police action, evaluate legitimacy and decide whether to comply, resist or escalate. This does not insulate these commitments from critique, contestation or failure in practice, but it does suggest that rights‐based democratic norms map onto the social psychological conditions under which order and conflict are actually produced.

Seen in this light, the cases analysed here can be read as the kind of historically grounded examples that critics such as Billig have long argued are necessary if social psychology is to avoid abstraction without consequence. They show how theory, once translated into institutional practice, does not merely interpret the social world but actively reshapes the conditions under which collective action, authority and legitimacy are enacted. The broader implication is not that social psychology can ever stand outside power, nor that co‐production offers a guaranteed route to ‘doing good’, but that the political purposes of psychological knowledge must be made more visible and governable rather than disavowed. Social and behavioural science is an institution of power, its concepts travel into the world, shaping institutional action and producing material consequences. By making these dynamics explicit, this paper argues for a social psychology that accepts its political entanglements and takes responsibility for the purposes to which its knowledge is put—reviving, in contemporary form, Lewin's injunction not only to produce good theory, but to use good theory to do good things.

## REFLEXIVITY AND POSITIONALITY STATEMENT

The ESIM‐oriented work referred to in this article has been conducted through sustained engagement with policing institutions and public‐order governance structures globally. Access to data and operational contexts has therefore been mediated through relations of power and shaped by the priorities and constraints of state authority. The work is not produced from a position external to power, nor does it claim political neutrality. It is generated within institutional settings that actively shape how collective action is governed.

The normative commitments underpinning this work are grounded in rights‐based democratic frameworks, including the European Convention on Human Rights and constitutional protections of assembly and expression in the United States. The aim is to contribute to policing practices that create conditions in which protest can occur safely and legitimately: where participants are protected from harm both by the state and by other crowd members; where collective action can challenge authority without being met by disproportionate force and where the application of law is bounded by legal frameworks designed to protect democratic freedoms. In this sense, the work is oriented not simply towards de‐escalation as a technical objective, but towards security achieved through proportionate, rights‐bounded policing in contexts of political contestation.

The Social Identity Approach and the Elaborated Social Identity Model are not presented as politically neutral frameworks, but neither were they derived to fit a pre‐given political programme. ESIM emerged from empirical research into the interactional dynamics of crowds and policing, and its convergence with rights‐based models of democratic governance reflects this underlying evidence base rather than prior normative design. The fact that ESIM‐based findings align with civic models of policing, legitimacy and consent is therefore not incidental, but an empirical consequence of analysing how conflict escalates and de‐escalates in practice. These alignments nevertheless remain politically consequential and contestable in their application. Dialogue‐ and legitimacy‐based approaches may be co‐opted as technologies of governance that stabilize authority without addressing deeper structural injustice. At the same time, the contemporary erosion and politicization of civic modes of policing—evident in the expansion of militarized public‐order doctrine, emergency powers and executive interventions in protest policing—underscore that such models cannot be taken for granted as stable features of democratic life. In this context, the decision to pursue co‐production with policing institutions reflects a situated political choice to defend and renew civic forms of policing grounded in consent, proportionality and rights‐based constraints on power. This work is therefore oriented not only towards harm reduction in specific operational settings, but towards sustaining the institutional conditions of democratic policing at a historical juncture when these conditions are increasingly under strain.

## AUTHOR CONTRIBUTIONS

**Clifford Stott:** Conceptualization; writing – review and editing; writing – original draft.

## CONFLICT OF INTEREST STATEMENT

None declared.

## Data Availability

Data sharing not applicable to this article as no datasets were generated or analysed during the current study.
